# Optimizing Biodegradable Poly(D,L-lactide) Scaffolds Reinforced with Graphene Oxide for Bone Tissue Regeneration

**DOI:** 10.3390/biomimetics10100700

**Published:** 2025-10-15

**Authors:** Esperanza Díaz, Ander García, Xabier León, Yolanda Merodio, Sylvie Ribeiro, Senentxu Lanceros-Méndez

**Affiliations:** 1 Departamento de Ingeniería Minera, Metalúrgica y Ciencia de Materiales, Escuela de Ingeniería de Bilbao, Universidad del País Vasco (UPV/EHU), 48920 Portugalete, Spain; 2 BCMaterials, Basque Centre for Materials, Applications and Nanostructures, UPV/EHU Science Park, 48940 Leioa, Spain; 3 Departamento de Organización de Empresas, Escuela de Ingeniería de Bilbao, Universidad del País Vasco (UPV/EHU), 48920 Portugalete, Spain; yolanda.merodio@ehu.eus; 4 CF-UM-UP—Physics Centre of Minho and Porto Universities and LaPMET—Laboratory of Physics for Materials and Emergent Technologies, University of Minho, 4710-057 Braga, Portugal; 5 IKERBASQUE, Basque Foundation for Science, 48009 Bilbao, Spain

**Keywords:** scaffolds, PDLLA, graphene oxide, mechanical properties, degradation, cytotoxicity

## Abstract

This study investigates the potential of porous poly(D,L-lactide) (PDLLA) scaffolds reinforced with graphene oxide (GO) for bone tissue engineering applications. Scaffolds were fabricated using thermally induced phase separation (TIPS) and characterized in terms of morphology, biodegradation, thermal and mechanical properties, and cytocompatibility. The incorporation of GO enhanced both mechanical strength and thermal stability, likely due to hydrogen bonding and electrostatic interactions between GO’s functional groups (carbonyl, carboxyl, epoxide, and hydroxyl) and PDLLA chains. In vitro degradation studies showed that GO accelerated degradation, while scaffolds with higher GO content retained superior mechanical strength. Cytotoxicity assays confirmed the biocompatibility of all scaffold variants, supporting their suitability for biomedical applications. Overall, the findings demonstrate how GO incorporation can modulate scaffold composition and performance. This provides insights for the design of improved systems for bone tissue regeneration.

## 1. Introduction

Autologous bone grafts remain a common strategy for treating bone defects and non-healing fractures; however, they are limited by donor site morbidity, restricted availability, and potential immune responses [1]. Consequently, considerable research has shifted toward alternative approaches involving mesenchymal stem cells and biomaterial scaffolds that promote osteogenic differentiation and tissue integration [2].

For bone regeneration, biomaterials must not only be biocompatible and biodegradable but also structurally mimic native bone. In particular, a porous and interconnected architecture is essential to facilitate cell migration, vascularization, and integration with surrounding tissues [2]. Graphene oxide (GO) has emerged as a promising additive due to its high surface area, abundant functional groups, and mechanical strength, which together enhance scaffold stability and cellular adhesion [3,4,5].

Successful bone tissue engineering further depends on scaffolds that meet critical parameters such as interconnected porosity above 70% and pore sizes between 100 and 500 µm, which are generally considered optimal for vascularization and osteogenic cell migration. Likewise, mechanical properties should approximate those of cancellous bone (2–12 MPa compressive strength and 0.1–2 GPa elastic modulus) to provide adequate stability without inducing stress shielding. Recent advances in polymer-based and composite scaffolds have reported enhanced strength and osteoconductivity through the incorporation of bioactive nanomaterials such as graphene oxide, hydroxyapatite, and bioactive glass. For instance, PDLLA/GO scaffolds have demonstrated improved compressive modulus and osteogenic activity compared to neat PDLLA [4], while HA/PDLLA composites have shown superior degradation profiles and cell adhesion properties [1].

Poly(D,L-lactide) (PDLLA) is a biodegradable polymer derived from lactic acid, consisting of a racemic mixture of L- and D-lactide. Its amorphous structure confers transparency, flexibility, and relatively rapid hydrolytic degradation, releasing lactic acid—a naturally occurring metabolite. These properties, together with its well-documented biocompatibility, have made PDLLA attractive for a range of biomedical applications [6,7], including optical fibers [7], drug delivery systems [8], nerve conduits [9], and tissue engineering scaffolds [10,11]. Importantly, PDLLA has supported the growth of diverse cell types, including osteoblasts and chondrocytes, further highlighting its relevance in regenerative medicine.

Recent studies indicate that combining PDLLA with GO can further expand its potential. For instance, GO has been incorporated into hydrogels for cartilage regeneration [12,13], improving mechanical properties and growth factor retention. In bone regeneration, GO is reported to promote osteogenic differentiation and enhance mechanical stability, although excessive concentrations may cause cytotoxicity, oxidative stress, and inflammation [5,14].

Taken together, these findings underscore the promise of PDLLA/GO composites for bone tissue engineering. Nevertheless, despite the growing interest in this system, systematic investigations that integrate degradation kinetics, mechanical performance, and cytocompatibility remain scarce. Existing studies often address these aspects separately, limiting the ability to draw comprehensive conclusions about scaffold suitability. To address this gap, the present work provides an in vitro evaluation that combines these key parameters under a unified framework, offering a clearer understanding of the potential of PDLLA/GO scaffolds in bone regeneration.

## 2. Experimental Details

### 2.1. Materials

The PDLLA polymer (high purity > 99.5%) was obtained from Byomer L900 (Bremen, Germany). The amorphous polymer was obtained by the polymerization of racemic lactide (a mixture of L- and D-lactide). The Mw was 150.000. Graphene oxide with an average particle size of 6–7 µm was supplied by Graphenea (San Sebastian, Spain). Monolayer content >95% under diluted conditions (in water, after sonication). The solvent 1,4-dioxane, used for scaffolds synthesis and phosphate-buffered saline (PBS) at a pH 7.2 and provided by Fluka Analytical (Sigma-Aldrich, Darmstadt, Germany), was employed for the in vitro degradation studies.

### 2.2. Scaffolds Fabrication

**Figure 1:**
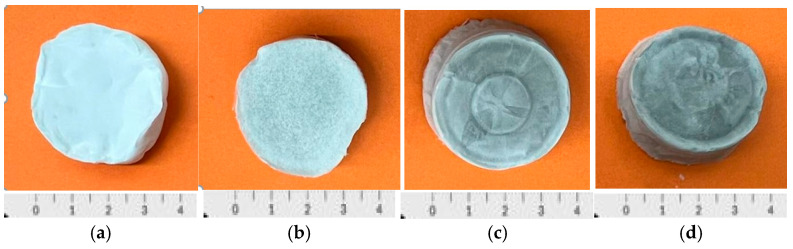
Macroscopic image of the different types of fabricated scaffolds: (**a**) PDLLA. (**b**) PDLLA/0.3% GO, (**c**) PDLLA/0.6% GO, (**d**) PDLLA/1% GO.

Porous scaffolds were fabricated using a 2.5% (*w*/*v*) solution of PDLLA in 1,4-dioxane. The solution was stirred at 50 °C for 2 h until the polymer fully dissolved, after which GO was dispersed using ultrasonic stirring with a probe sonicator (20 kHz, 30–40% amplitude) in pulsed mode (2 s ON/4 s OFF. The mixture, containing varying GO proportions relative to the total polymer mass (0%, 0.3%, 0.6%, 1%), was freeze-dried using a LyoQuest system (Telstar, Barcelona, Spain) for 7 days. Porosity was determined using Thermo Fisher’s Avizo software (Thermo Fisher Scientific, Waltham, MA, USA). Porous scaffolds with up to 90% porosity were successfully obtained through this process, as shown in Figure 1.

### 2.3. Samples Characterization, In Vitro Degradation, and Cytotoxicity Assessment

All experiments were performed in quintuplicate (*n* = 5) for each sample and time point. To ensure data consistency and minimize experimental variability, individual measurements that deviated more than 5% from the preliminary mean value were considered outliers and excluded. The corresponding test was then repeated to maintain a complete set of five replicates per condition.

Prior to morphological examination, the samples were coated with a thin layer of gold using a JEL Ion Sputter JFC-1100 (Amiron Machinery, Oxnard, CA, USA), operating at 1200 V and 5 mA. The morphology of the scaffolds was then analysed using a Scanning Electron Microscope (SEM) (HITACHI S-3).

The thermal characteristics of the scaffolds were assessed through differential scanning calorimetry (DSC), using equipment from TA Instruments (Waters, NC, USA). The analysis was performed under a nitrogen atmosphere to prevent oxidation. The samples were heated from 0 °C to 250 °C at 10 °C/min (1st run), then cooled at 10 °C/min, and finally heated at 10 °C/min (3rd run). Below 0 °C there is no appreciable transformation for our scaffolds.

Compression tests were performed using an Instron universal testing machine equipped with a 100 kN load cell. A constant testing speed of 1 mm.min^−1^ was applied throughout all experiments. The tests were designed to achieve 90% compression; although in certain instances, the equipment’s maximum load capacity was reached before achieving the full-intended compression. The cylindrical samples used for testing presented a diameter of 35 mm and a height of approximately 10 mm. The stiffness of the scaffolds was determined by calculating the slope of the initial linear region of the stress–strain curve.

For the in vitro degradation studies (ISO 13781 [15], the scaffolds were cut into rectangular samples measuring 0.5 cm^2^, and their initial weights were recorded to evaluate in vitro degradation in Phosphate-Buffered Saline (PBS) at 37 °C cover time periods of 0, 18, 36, 54 and 72 days. A PCE 228 pH meter (PCE Instruments, Pons, Alicante, Spain) was employed to monitor pH variations in the PBS prior to degradation. Mass loss and water absorption were calculated using Equations (1) and (2):

(1) $$\mathrm{WL}\;\%=\frac{Wo-Wr}{Wo}\times 100$$

(2) $$Wa\;\%=\frac{Ww-Wr}{Wr}\times 100$$

where WL % represents the percentile weight loss and *Wa %* denotes the water absorption. *Wo* is the initial mass before degradation, *Wr* is the weight of the dry sample after degradation, and *W_W_* is the weight of the sample after degradation with surface water removed.

An assessment of indirect cytotoxicity was carried out following a modified protocol based on the ISO 10993-5 standard. Membranes with a concentration of 0.1 g·mL^−1^ were cut into suitable dimensions and subjected to UV sterilization for 2 h, ensuring 1 h of exposure per side. After sterilization, the samples underwent three consecutive washes with phosphate-buffered saline (PBS), each lasting 5 min, to eliminate any solvent residues.

For the preparation of the conditioned medium, the sterilized samples (0.1 g·mL^−1^) were immersed in a 24-well polystyrene tissue culture plate, containing Dulbecco’s Modified Eagle Medium (DMEM) supplemented with 1 g·L^−1^ glucose (Gibco), 10% fetal bovine serum (FBS) (Biochrom), and 1% penicillin/streptomycin (P/S) (Biochrom). The samples were incubated at 37 °C in a humidified environment with 5% CO_2_ for 24 h. A 20% dimethyl sulfoxide (DMSO) (Sigma-Aldrich) solution served as the positive control, while fresh cell culture medium (without sample exposure) was used as the negative control.

Simultaneously, MC3T3-E1 cells were seeded into a 96-well polystyrene tissue culture plate at a concentration of 3 × 10^4^ cells·mL^−1^ and incubated for 24 h to allow cell adhesion. After this period, the existing culture medium was carefully discarded, and 100 µL of conditioned medium from the pre-incubated samples was added to each well. The cells were then maintained under identical incubation conditions for additional 72 h.

To evaluate cell viability, the MTS assay (3-(4,5-dimethylthiazol-2-yl)-5-(3-carboxymethoxyphenyl)-2-(4-sulfophenyl)-2H-tetrazolium) was employed. At predefined time points, MTS reagent was mixed with DMEM in a 1:5 ratio and added to each well, followed by 2 h incubation at 37 °C. Absorbance readings were recorded at 490 nm using a microplate reader. The experimental results, derived from four replicate per sample and control, were expressed as the mean cell viability percentage ± standard deviation (SD). The percentage of viable cells was calculated using Equation (3):

(3) $$Cell\;viability\;\left(\%\right)=\frac{Absorbance\;of\;sample}{Absorbance\;of\;negative\;control}\times 100$$

## 3. Results and Discussion

### 3.1. Morphology

Thermally Induced Phase Separation (TIPS) was applied to PLA scaffolds by Schugens et al. [16,17]. It involves inducing a solid–liquid or liquid–liquid phase separation by dissolving the polymer in a solvent and cooling the solution to a specific temperature. The cooling causes phase separation into a polymer-rich phase and a polymer-poor phase. The solvent is then removed by freeze-drying. The main advantage of the phase separation method is that the morphology and orientation of the pores can be adjusted to the thermodynamic and kinetic parameters of the process. Its disadvantages include the use of potentially toxic solvents (1,4 dioxane) and a high degree of porosity anisotropy. The latter can be beneficial for certain biomedical and industrial applications such as nerve and bone regeneration due to mechanical cushioning [18,19].

Scanning Electron Microscopy (SEM) is a valuable tool for analyzing scaffold degradation because it provides high-resolution visualization of surface morphology and microstructural changes over time. As the scaffold degrades, SEM allows for the observation of features such as pore collapse, surface erosion, crack formation, and fiber fragmentation. These morphological alterations are closely linked to changes in mechanical properties, mass loss, and cellular interactions. By capturing detailed images at different degradation stages, SEM helps to correlate structural integrity with functional performance, offering critical [20].

**Figure 2:**
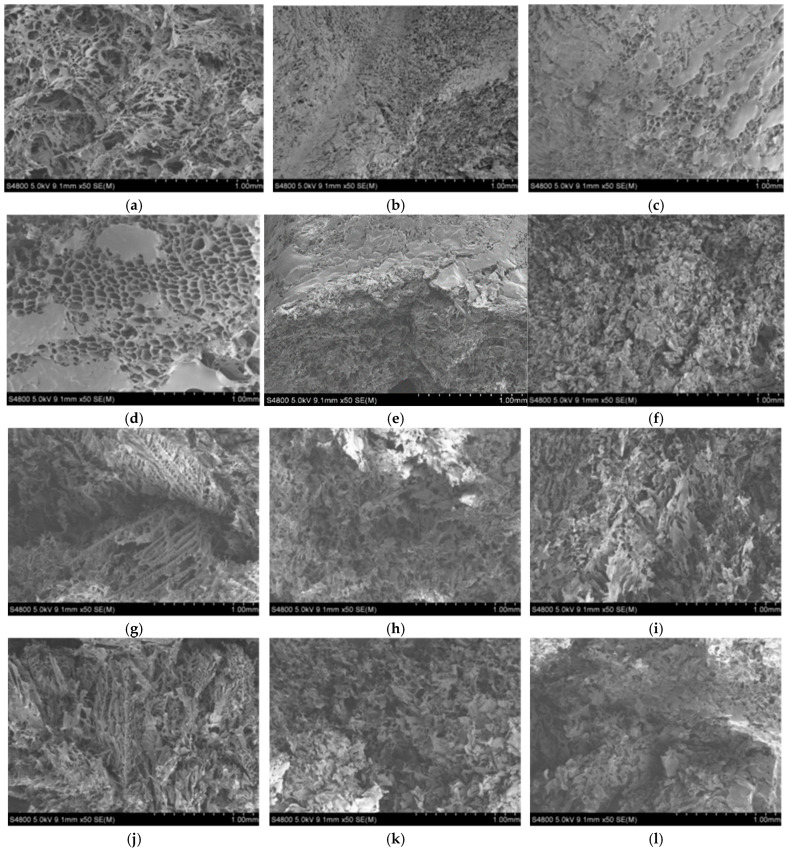
SEM images showing the surface morphology of PDLLA/ GO scaffolds at different degradation stages. (**a**) PDLLA before degradation. (**b**) PDLLA after in vitro degradation for 54 days. (**c**) PDLLA after in vitro degradation for 72 days. (**d**) PDLLA/0.3% GO before degradation. (**e**) PDLLA/0.3% GO after degradation for 54 days. (**f**) PDLLA/0.3% GO after degradation for 72 days (**g**) PDLLA/0.6% GO before degradation. (**h**) PDLLA/0.6% GO after degradation 54 days. (**i**) PDLLA/0.6% GO after degradation for 72 days. (**j**) PDLLA/ 1% GO before degradation. (**k**) PDLLA/ 1% GO after degradation for 54 days. (**l**) PDLLA/ 1% GO after degradation for 72 days.

TIPS produced highly porous scaffolds as can be observed in the representative SEM images provided in Figure 2. The effect of scaffold composition and degradation can be clearly seen in Figure 2.

TIPS successfully generated highly porous scaffolds with tunable morphology. The incorporation of GO significantly influenced pore size and organization. At low concentration (0.3%), scaffolds exhibited a more compact and aligned morphology, while higher GO loadings (0.6–1%) produced irregular structures with reduced pore size, likely due to changes in phase separation dynamics and crystallization kinetics. These observations align with previous findings where GO-modified PLLA scaffolds exhibited altered microarchitecture, attributed to GO’s high surface energy and its ability to act as a nucleating agent [20,21,22].

From a biomimetic perspective, the anisotropic tubular structures promoted by GO resemble natural bone’s hierarchical porosity, which provides both mechanical strength and pathways for vascularization. Similar biomimetic designs have been reported in collagen/HA scaffolds, where anisotropy improved osteoblast alignment and nutrient diffusion [23]. Compared to such systems, GO offers an additional advantage by enhancing mechanical reinforcement without compromising cytocompatibility, although pore collapse during degradation remains a limitation.

### 3.2. pH Variation

pH measurement during scaffold degradation is essential to monitor the chemical environment resulting from material breakdown. As scaffolds degrade (particularly those made from biodegradable polymers) they may release acidic or basic byproducts, which can alter the local pH of the surrounding medium. Changes in pH can significantly affect cell viability, enzyme activity, and the overall biocompatibility of the scaffold [24].

**Figure 3:**
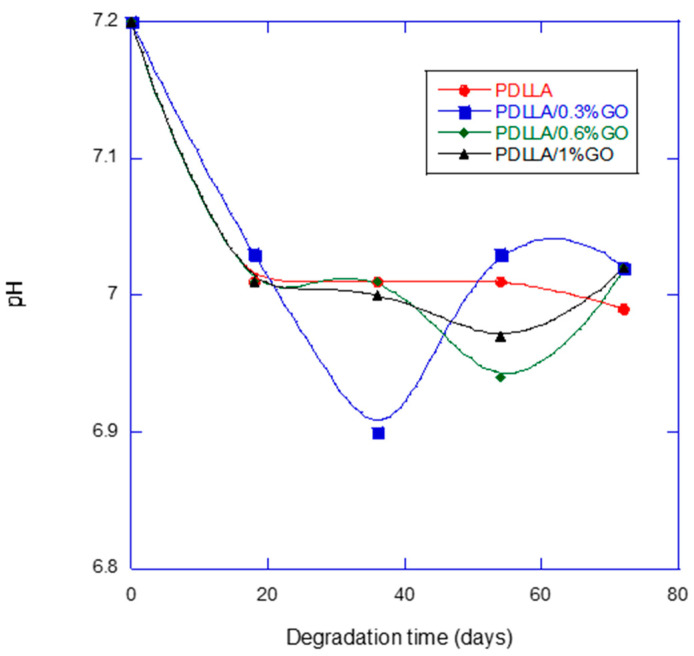
pH variation in the Phosphate-Buffer Solution for PDLLA and PDLLA/GO scaffolds as a function of degradation time.

In Figure 3, a notable observation was the minimal pH decrease (from 7.2 to 7.0) over 72 days, showing significant differences between GO-reinforced and neat PDLLA scaffolds. We consider that, although the absolute pH variation seems small, it is relevant in a biological context, since even small decreases can influence enzyme activity and cellular response. Furthermore, the fact that the time to reach the minimum pH value differs between groups shows that the presence of GO not only modulates the magnitude of the variation, but also the kinetics of the degradation process, which supports our interpretation of the regulatory effect of GO on the microenvironment. This is particularly important given the typical acidification of polyesters such as PLA, whose degradation into lactic acid may provoke local inflammation and reduce biocompatibility [24,25].

While some authors have reported that GO accelerates the hydrolytic degradation of PLLA, attributed to the hydrophilic functional groups of GO that promote water infiltration and ester bond hydrolysis [26], others have found that GO in electrospun PLA membranes supports a more favorable microenvironment, maintaining an appropriate pH for nerve regeneration [27]. These contrasting behaviors likely depend on scaffold architecture, dispersion methods, and degradation conditions.

The observed pH stability can be interpreted as a biomimetic feature, since native tissues tightly regulate extracellular pH to preserve enzymatic activity and cell viability. Maintaining a near-neutral microenvironment minimizes inflammatory responses and better mimics physiological homeostasis. GO-enhanced hydrophilicity may provide a balanced water diffusion into the polymer matrix, preventing the rapid accumulation of acidic byproducts [28].

### 3.3. Water Absorption

The variation in water uptake by a scaffold during in vitro degradation generally affects several critical parameters of the system. Increased water absorption can accelerate hydrolytic degradation, alter the scaffold’s mechanical integrity, and influence the release kinetics of embedded bioactive agents. Moreover, water uptake can impact the scaffold’s porosity, swelling behavior, and microstructural stability, which in turn modulate cell adhesion, proliferation, and overall biocompatibility [27].

**Figure 4:**
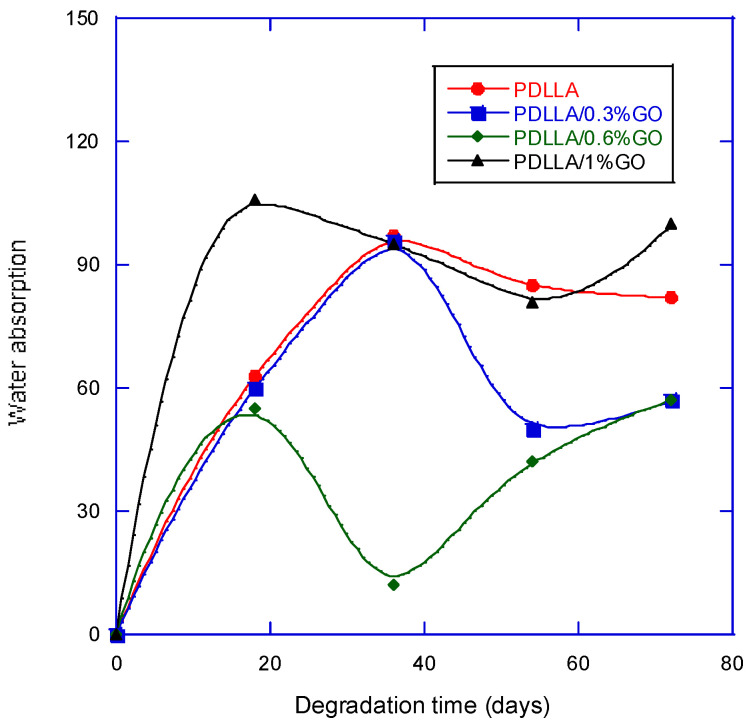
Water absorption, for PDLLA and PDLLA/GO scaffolds, as a function of degradation time.

The findings (Figure 4) demonstrate that GO significantly enhances scaffold hydrophilicity, leading to increased water uptake during the early stages of degradation, followed by a stabilization phase as oligomer dissolution progresses.

GO contains abundant oxygenated functional groups (hydroxyl, epoxy, carboxyl, and carbonyl), which impart hydrophilic character and increase scaffold–water interactions. As can be seen in Figure 4, scaffolds with 1% GO exhibited greater water absorption during the first 18–36 days compared with neat PDLLA. This is in agreement with previous reports that the presence of GO enhances hydrophilicity and accelerates water penetration into PLA matrices [27,28]. Such behavior can facilitate hydrolytic attack on ester bonds, thereby influencing degradation kinetics.

The early increase in water absorption, particularly at higher GO contents, reflects the dual role of GO: (i) providing hydrophilic domains that promote water influx, and (ii) creating interfacial regions within the polymer that act as diffusion pathways [29]. These effects are consistent with studies on GO-reinforced PLLA and PLGA scaffolds, where GO accelerated initial water uptake and triggered earlier onset of hydrolysis [29,30].

Interestingly, after the initial absorption phase, water uptake decreased and stabilized as degradation progressed. This transition may be explained by the release of soluble oligomers into the medium, which reduce water absorption capacity by balancing osmotic forces and altering scaffold porosity. Similar biphasic water uptake patterns have been reported in PLA/GO composites and in other polyester-based biomaterials, where oligomer release and structural compaction modulate fluid transport [31,32].

From a biomimetic standpoint, this staged absorption–stabilization behavior mirrors certain aspects of extracellular matrix (ECM) remodeling, where early fluid influx supports enzymatic activity and matrix turnover, followed by stabilization as equilibrium is re-established. Such behavior may be advantageous for tissue engineering scaffolds, since controlled water uptake influences both mechanical stability and the local microenvironment for cell survival.

Enhanced hydrophilicity is generally favorable for cell adhesion and proliferation, as hydrophilic surfaces improve protein adsorption and integrin-mediated interactions [33]. However, excessive water absorption can compromise mechanical integrity by accelerating bulk degradation. The balance observed here, with initial hydrophilicity followed by stabilization, suggests that GO–PDLLA scaffolds may provide a transiently permissive environment for early cell colonization while preserving long-term scaffold structure.

### 3.4. Mass Loss

Mass loss measurement in a scaffold is performed to evaluate its degradation behavior over time. This parameter provides quantitative insight into how the scaffold breaks down under physiological or simulated conditions. Tracking mass loss helps assess the rate and extent of material degradation, which is critical for ensuring that the scaffold supports tissue regeneration without premature structural failure or excessive persistence [18].

**Figure 5:**
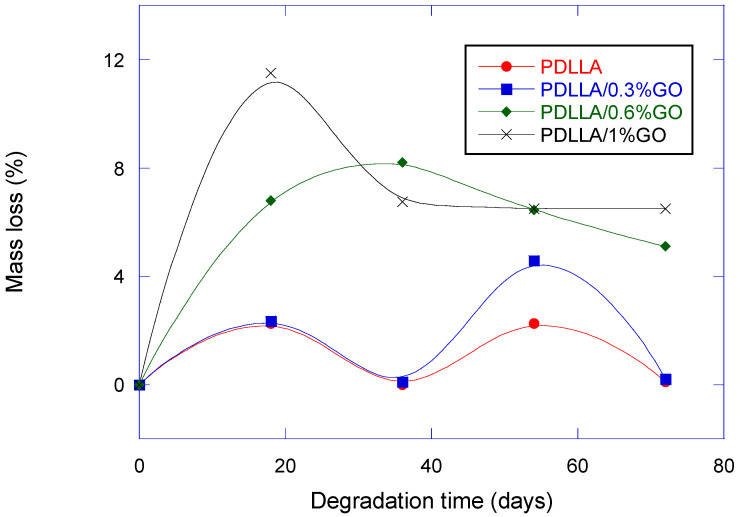
Mass loss of PDLLA and PDLLA/GO scaffolds as a function of degradation time.

Figure 5 presents the mass loss as a function of degradation time. Higher GO content (0.6–1%) produced rapid initial weight loss, while lower GO content (0–0.3%) degraded more gradually. This finding is consistent with prior studies where GO’s oxygenated groups accelerated ester hydrolysis [18,31,32,33].

Biomimetically, accelerated degradation parallels the turnover of trabecular bone, which undergoes rapid remodeling. Conversely, gradual degradation is more akin to cortical bone, where scaffolds must persist longer to support load-bearing. Compared with ceramic-based scaffolds (which often degrade too slowly), GO-containing scaffolds offer tunability that better mirrors natural bone’s remodeling rates [34,35,36].

### 3.5. Thermal Analysis

The glass transition temperature (Tg) is measured to assess changes in the thermal and mechanical properties of the scaffold during degradation. Tg represents the temperature at which a material transitions from a rigid, glassy state to a more flexible, rubbery state. As degradation progresses (especially in polymeric scaffolds) molecular weight decreases and plasticization by water uptake can lower the Tg. Monitoring Tg provides insights into the structural integrity, processability, and mechanical performance of the scaffold over time [37].

**Figure 6:**
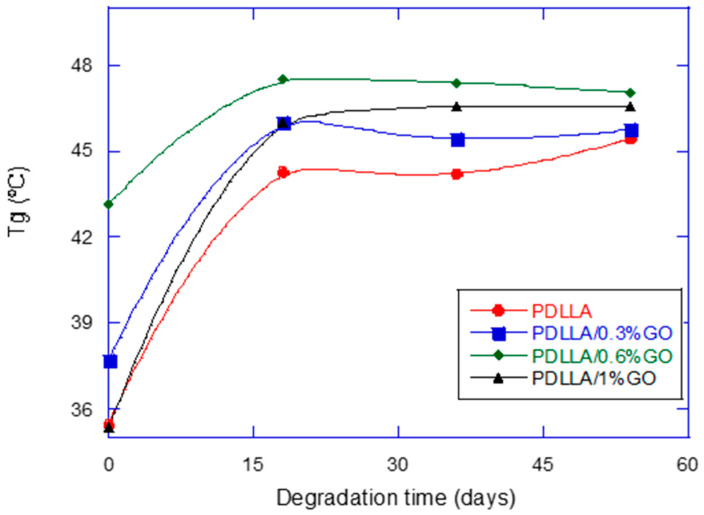
Glass transition temperature (°C) of PDLLA and PDLLA/GO scaffolds as a function of degradation time.

The incorporation of graphene oxide (GO) influenced the thermal behavior of the scaffolds, as shown in Figure 6. The addition of GO led to an increase in the glass transition temperature (Tg), suggesting restricted polymer chain mobility due to polymer–filler interactions. Similar reinforcement effects have been widely reported in other GO–polymer composites [36,37,38]. Interestingly, the scaffold containing 1% GO exhibited a lower Tg compared with those at intermediate concentrations, most likely as a result of GO agglomeration and structural defects. Graphene oxide (GO) tends to agglomerate due to π–π interactions and hydrogen bonding, which may hinder homogeneous dispersion. This intrinsic behavior can lead to irregular distribution within the scaffold and thus account for deviations from the expected results [35]. This observation emphasizes the importance of achieving a homogeneous GO dispersion to fully exploit its reinforcing potential.

From a biomimetic standpoint, the observed increase in Tg reflects enhanced thermal stability under in vitro conditions, which is desirable for maintaining scaffold functionality during early implantation stages. Natural bone exhibits comparable resilience, derived from the synergistic interplay between its organic matrix and inorganic mineral phase. When compared with other common fillers such as bioactive glass, GO offers reinforcement with superior nanoscale dispersibility and flexibility in tuning physicochemical interactions [39].

### 3.6. Mechanical Properties

Mechanical properties are measured to evaluate the scaffold’s ability to maintain structural support throughout the degradation process. Parameters such as compressive strength, tensile strength, and elastic modulus are critical for ensuring that the scaffold can withstand physiological loads and provide a stable environment for cell attachment and tissue regeneration. As degradation progresses, mechanical properties typically deteriorate due to material erosion, loss of molecular weight, and changes in porosity. Monitoring these properties allows for the prediction of scaffold performance in vivo and ensures that the degradation profile aligns with the rate of new tissue formation, which is essential for successful integration and function in tissue engineering applications.

Scaffolds intended for bone tissue engineering must combine adequate porosity with sufficient mechanical resistance to endure physiological stresses until new tissue is formed. A critical aspect is therefore the balance between mechanical stability and controlled degradation, since premature loss of integrity could compromise their clinical performance.

**Figure 7:**
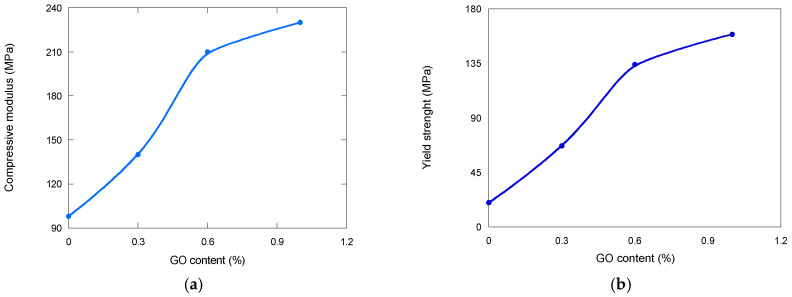
(**a**) Compressive modulus and (**b**) yield strength of PDLLA and PDLLA/GO scaffolds as a function of GO content.

**Figure 8:**
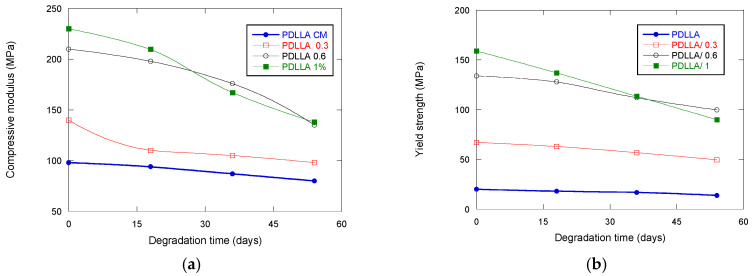
(**a**) Compressive modulus and (**b**) yield strength of PDLLA and PDLLA/GO scaffolds as a function of degradation time.

Our results show (Figure 7 that the incorporation of graphene oxide (GO) markedly enhances the mechanical behavior of the scaffolds. Increasing GO content from 0 to 1% produced a substantial rise in compressive modulus (from 98 to 230 MPa, an increase of 2.34%) and yield strength (from 20 to 159 MPa, an increase of 7.95%) (Figure 8). This reinforcement effect is attributed both to the intrinsic stiffness of GO and to the concomitant reduction in pore size. Importantly, the compressive modulus values achieved with higher GO concentrations fall within the mid-range of those reported for human cortical bone [32], highlighting their potential suitability for load-bearing applications. However, excessively high stiffness may alter the stress distribution at the scaffold–bone interface, potentially inducing stress-shielding phenomena. Thus, the tunability of GO content offers a valuable tool to optimize scaffold mechanics depending on the target anatomical site.

Degradation studies revealed contrasting trends (Figure 8). While scaffolds without GO preserved their mechanical properties almost unaltered over time, the presence of GO accelerated degradation. This effect may be advantageous for promoting faster tissue turnover in clinical scenarios requiring rapid resorption, but it also raises concerns about long-term stability. Notably, scaffolds with 0.6% and 1% GO maintained mechanical properties superior to pure PDLLA even after degradation, indicating that the reinforcing effect of GO compensates for the accelerated resorption, at least during the early and intermediate stages. These findings align with previous studies [37,38,39], reinforcing the notion that GO incorporation can be tailored to achieve a balance between structural integrity and resorbability.

In summary, the addition of GO provides a dual benefit: initial mechanical reinforcement and degradation-mediated tunability. The fact that the mechanical properties reach values comparable to natural bone underscores the biomimetic potential of these scaffolds. Nevertheless, further in vivo studies are necessary to confirm whether the accelerated degradation induced by GO favors or hinders functional bone regeneration in different clinical contexts.

### 3.7. Cytotoxicity Assays

**Figure 9:**
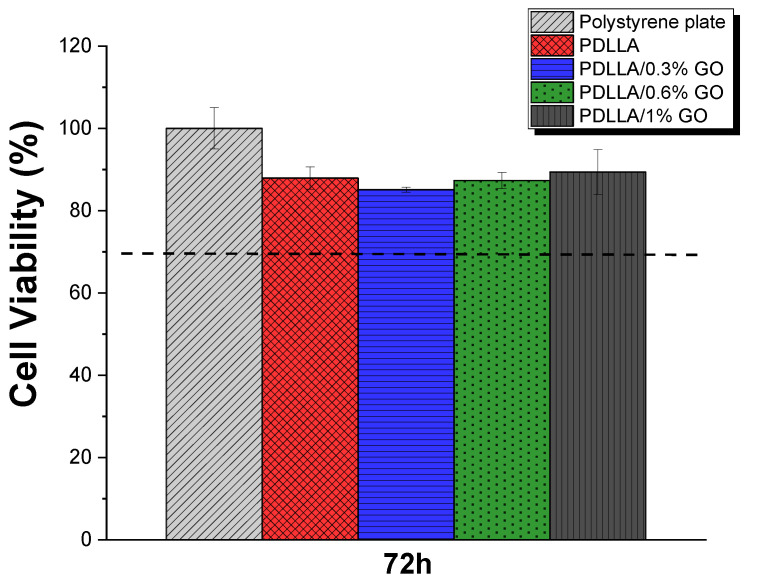
Cell viability of MC3T3-E1 cells exposed to conditioned media from different sample groups over a 72 h period.

The cytotoxic response of the scaffolds was evaluated by assessing the metabolic activity of MC3T3-E1 cells cultured with conditioned media from PDLLA samples containing different GO concentrations. Cell viability was quantified using the MTS assay after 72 h (Figure 9). Although a modified ISO 10993-5 method was applied in this study, the reference threshold commonly used to indicate cytotoxicity—a reduction in cell viability greater than 30%—was considered to contextualize the results.

As shown in Figure 9, none of the tested groups exhibited cytotoxic effects, as cell viability remained well above the critical threshold. These results align with previous studies on GO-reinforced polymeric scaffolds, in which no significant cytotoxicity was detected. Supporting evidence from the literature further substantiates these findings. For example, PDLLA scaffolds incorporating super-hydrophilic blends of graphene and multi-walled carbon nanotubes have been reported to enhance osteoblast function in vitro and promote bone regeneration in vivo without detectable cytotoxicity [40]. Similarly, recent reviews on graphene-based biomaterials emphasize their strong potential for tissue engineering applications, particularly when combined with biodegradable polymers such as PDLLA, with no reported adverse cytotoxic effects [41]. Furthermore, scaffolds composed of GO and PLLA demonstrated non-cytotoxic behavior toward human dental pulp stem cells, while simultaneously promoting adhesion and proliferation [42].

Taken together, these findings confirm that the incorporation of GO into PDLLA scaffolds does not compromise cell viability and may even provide a favorable microenvironment for bone tissue regeneration. This excellent biocompatibility, combined with the mechanical reinforcement and tunable degradation described in previous sections, positions GO/PDLLA scaffolds as highly promising candidates for translational applications in bone tissue engineering.

## 4. Conclusions

This study demonstrates that the incorporation of graphene oxide (GO) into poly(D,L-lactic acid) (PDLLA) scaffolds results in multifunctional biomaterials with enhanced properties suitable for bone tissue engineering. The GO/PDLLA scaffolds exhibited improved mechanical performance and tunable degradation profiles without compromising biocompatibility. In vitro cytotoxicity assays confirmed that none of the tested formulations reduced cell viability below the ISO 10993-5 threshold, indicating excellent cytocompatibility across all GO concentrations.

Furthermore, the combined effects of structural reinforcement, controlled degradation, and maintenance of a neutral pH microenvironment highlight the potential of GO as a functional additive for PDLLA-based scaffolds. These features may contribute to creating a favorable environment for osteoblast adhesion, proliferation, and differentiation, ultimately supporting bone regeneration.

Taken together, our findings position PDLLA/GO scaffolds as promising candidates for translational applications in bone tissue engineering, bridging the gap between current biodegradable polymer systems and next-generation, mechanically robust, and biologically supportive scaffolds. Future work should focus on in vivo validation and long-term performance assessments to confirm their clinical potential.

## Data Availability

Additional data will be provided upon request.
